# Supplementary material for: Microbial methane cycling in a landfill on a decadal time scale
Source: Nat Commun. 2023 Nov 16;14:7402. doi: 10.1038/s41467-023-43129-x (PMC10654671; doi:10.1038/s41467-023-43129-x)
Supplement: Supplementary file 1 — Supplementary Information [file 41467_2023_43129_MOESM1_ESM.pdf]

Supplementary Information for
Microbial methane cycling in a landfill on a decadal time scale

Daniel S. Grégoire, Nikhil A. George, Laura A. Hug

Laura A Hug, Daniel S. Grégoire
Email: laura.hug@uwaterloo.ca, danielgregoire@cunet.carleton.ca

This PDF file includes:

Supplementary Notes 1 to 4
Figures S1 to S13
Table S1 and S2
Supplementary References

Supplementary Note 1: BLAST search for *mtaA/mtsA* in *Methanofastidiosales* genomes

In classifying the methanogenic capabilities observed in putative methanogen MAGs, we noted DRAM's product file showed that MAG STF1_149 from the order *Methanofastidiosales* had no genes demonstrating the potential to access inorganic or organic substrates to support methane production despite having an annotated *mcrA* gene (Fig 2). The first genomic characterization of the *Methanofastidiosales* inferred that members of this order carry out methanogenesis in a fastidious manner via the reduction of methylated thiols thanks to the *mtsA* gene coding a methyltransferase specific to methylthiol-bearing compounds [8].

To determine whether MAG STF1_149 from the order *Methanofastidiosales* could potentially support methanogenesis using the reduction of methylthiols via the *mtsA* gene, we conducted an in-depth analysis on this genome and others from the order *Methanofastidiosales* retrieved from GTDB. To confirm that DRAM could annotate the *mtsA* gene, we searched the raw annotations for all MAGs obtained from the landfill. We confirmed that the *mtsA* gene was annotated in 3 MAGs (i.e., STD2_188, STF1_134, and STF2_199) [see Supplementary Data 7 on the Open Science Framework (OSF) at DOI 10.17605/OSF.IO/6X5ZC]. Notably, DRAM failed to annotate the *mtsA* gene for MAG STF1_149 [see Supplementary Data 7 on the Open Science Framework (OSF) at DOI 10.17605/OSF.IO/6X5ZC]. Although failure to annotate the *mtsA* gene in MAG STF1_149 could be attributable to the low completion of this genome [see Supplementary Data 5 on the Open Science Framework (OSF) at DOI 10.17605/OSF.IO/6X5ZC], the *mtsA* gene could not be annotated in any publicly available *Methanofastidiosales* genomes even when including the UniRef90 database to improve annotations (data not shown).

The *mtsA*-encoding MAGs, STD2_188, STF1_134, and STF2_199, were all taxonomically classified to the species *Methanosarcina* sp.002499445 within the order *Methanosarcinales* [see Supplementary Data 1 on the Open Science Framework (OSF) at DOI 10.17605/OSF.IO/6X5ZC]. Furthermore, 2 KEGG IDs were presented side by side for these annotations: K16954 for the *mtsA* gene (https://www.genome.jp/dbget-bin/www_bget?ko:K16954), and K14080 for the *mtaA* gene (https://www.genome.jp/entry/K14080) (see Supplementary Data 7 on the Open Science Framework (OSF) at DOI 10.17605/OSF.IO/6X5ZC).

The *mtaA* gene is a methyltransferase that catalyzes the final step of methylotrophic methanogenesis and is thought to be specific to methanol and/or amine molecules, whereas *mtsA* is thought to be specific to methylated thiols [8–10]. This dual annotation suggests that DRAM is unable to distinguish between these two homologous genes using sequences available through reference databases such as KEGG, Uniref90, and Pfam. When verifying the literature references associated with the KEGG entry K16954 denoting the *mtsA* gene, both references associated with this entry are studies on *Methanosarcina barkeri* (references accessed via https://www.genome.jp/dbget-bin/www_bget?ko:K16954) and the taxonomy associated with the genes that KEGG refers to are dominated by members of the *Methanosarcina* genus. These observations suggest that DRAM's capacity to annotate *mtaA*/*mtsA* genes and potential homologs relies on sequences that are divergent from the *mtaA*/*mtsA* sequence of members of the *Methanofastidiosales*.

To identify putative *mtaA*/*mtsA* homologs in the *Methanofastidiosales* MAG STF1_149, we retrieved the *mtsA* sequence from the *Methanofastidiosum methylophilus* genome (via https://www.ncbi.nlm.nih.gov/protein/KYC51554.1?report=fasta) and performed a BLAST

search using MAG STF1_149 as the searchable database. This search indicated that MAG STF1_149 harboured a gene with similarity score of 67% to the *mtsA* sequence of *Methanofastidiosum methylophilus*, which was annotated as a uroporphyrinogen decarboxylase by DRAM (corresponding to Pfam entry PF01208 shown on https://www.uniprot.org/uniprotkb/Q48924/entry) (see Supplementary Data 7 on the Open Science Framework (OSF) at DOI 10.17605/OSF.IO/6X5ZC). Notably, the same Pfam entry applies to the *mtaA* gene, further supporting that DRAM lacks the ability to distinguish between *mtaA* and *mtsA*. The similarity score being close to 70% further suggests that this gene in MAG STF1_149 may carry out the same function as *mtaA/mtsA* but may have diverged due to a specialized methanogenic lifestyle optimized for methylthiol-bearing compounds.

As an additional test for database biases limiting our ability to annotate *mtaA/mtsA* homologs in *Methanofastidiosales* genomes, the putative *mtaA/mtsA* sequence detected in MAG STF1_149 was used as input for a tblastn search to identify the taxa with the best hit. The best hit for the hypothetical *mtsA* gene in MAG STF1_149 had a similarity score of 68% and was associated with a sequence from *Methanosarcina acetivorans* C2A (accession AE010299.1, https://www.ncbi.nlm.nih.gov/nucleotide/AE010299.1?report=genbank&log$=nucltop&blast_rank=1&RID=V1FJSSNJ016). Notably, no sequences from the *Methanofastidiosales* order showed up in the alignment table, despite the similarity score being nearly identical to those obtained with a BLAST search of MAG STF1_149 using the *mtsA* sequence from *M. methylophilus* as a query sequence.

Regardless of whether the gene of interest in MAG STF1_149 represents a homolog for *mtaA* or *mtsA,* this result demonstrates that DRAM may not have been able to effectively annotate divergent sequences of this gene because it is referencing sequences in KEGG and Pfam

originally obtained from the *Methanosarcina* genus. The inability to detect the *mtsA* gene with tools such as DRAM also suggests that divergent pathways for methanogenesis that rely on methylthiols remain overlooked in the methane cycle and merit further investigation. We conclude that there is evidence, albeit inconclusive, that MAG STF1_149 can carry out methanogenesis via a divergent *mtsA* homolog given that all other machinery to access known substrates for methanogenesis are absent.

Supplementary Note 2: Assessing the relative abundance of putative acetogens

We used the distribution of putative acetogens as a proxy to assess the potential for syntrophs to supply known substrates to methanogens in municipal solid waste (MSW). Although this analysis does not capture all potential metabolic pathways that could supply carbon substrates to methanogens, such a characterization falls outside of the scope of the current study.

Our metabolic modeling of putative acetogens showed that almost all the 456 MAGs identified as putative acetogens fell into category iv) MAGs that carried the genes coding for the phosphotransacetylase and acetate kinase (see Methods). Only 27 of the 456 MAGs had 6/7 steps required for the Wood-Ljungdahl (WL) pathway but lacked an annotated phosphotransacetylase and acetate kinase. Only 3 of the 456 MAGs had a complete carbonic anhydrase/acetyl-CoA synthase pathway (CODH/ACS) but these MAGs lacked an annotated phosphotransacetylase and acetate kinase (Fig S6). This observation suggests that proton reducing acetogens are not major acetate generators in this habitat and are potentially outcompeted by methanogens for key metabolic substrates (e.g., hydrogen or carbon dioxide).

We observed that landfill cells E, F1, and F2 tended to have more putative acetogenic MAGs at relative abundances ranging from 5 to 10% (Fig S6). These relative abundance values exceeded the mean and median values at the whole community level suggesting putative acetogens are abundant in habitats that are supporting high methane production (Fig 1and Fig S6). The relative abundance of putative acetogens was ~3% for cells D1 and D2 and closer to the mean and median values for the whole community, which aligns with these being lower methane production habitats (Fig 1 and Fig S6). Cell C displayed the most abundant putative acetogens, which were also the dominant MAGs detected at the whole community scale (Fig S6). Among the older landfill cells, cell C displayed more varied pathways that could potentially support methanogenesis, suggesting these acetogens may be suppling substrates to continue supporting slow methane production in ageing landfills (Fig 3 and Fig S6). Putative acetogens were not abundant or diverse in cells A and B, suggesting there is limited capacity to generate known substrates to support methanogenesis, which aligns with our geochemical and microbiological observations (Fig S6).

Supplementary Note 3: Putative methanotrophs in the *Nevskiaceae*, *Acetobacteraceae*, and *Mycobacteriaceae*

In the *Nevskiaceae* family, four genomes had all genes required for the pMMO complex (i.e., MAG STE_114 from this study and its close relative CAU-1509 sp. 005047655, *Panacagrimonas perspica*, and *Polycyclovorans* sp.002706265) and *Solimonas aquatica*'s genome had the full gene repertoire for both the pMMO and sMMO complexes (Fig S10). In the *Acetobacteraceae* family, four genomes carried the genes required for the sMMO complex (i.e., MAGs STC_13 and STB_66 from this study, which are closely related to each other, *Rhodopila*

sp. 903915725 and *Rhodopila* sp. 903851415; Fig S11). In the *Mycobacteriaceae* family, which has substantially more publicly available genomes than the other two families examined, two genomes carried the genes required for the pMMO complex (i.e., *Mycobacterium dioxanotrophicus* and *Smaragdicoccus niigatensis*), five genomes carried the genes required for the sMMO complex (i.e., MAG STB_95 from this study, *Mycobacterium moriokaense* A, *Mycobacterium* sp. 003719305, *Mycobacterium holsaticum*, and *Mycobacterium pulveris*), and four genomes carried the genes required for both pMMO and sMMO (i.e., *Mycobacterium* sp. 002887815, *Mycobacterium* sp. 003053865, *Mycobacterium chubuense* A, and *Mycobacterium rhodesiae A*; Fig S12). We note MAG STB_95 from this study was most closely related to *Mycobacterium* sp.007714185, which did not encode genes for methanotrophy (Fig S12).

The detection of putative methanotrophs in the *Mycobacteriaceae* merits additional context. Over the last 70 years, a handful of studies have sporadically provided direct and indirect evidence of methanotrophy in strains identified as members of the genus *Mycobacterium* [11–13]. This conflicting evidence may explain why members of *Mycobacteriaceae* have been precluded from recent surveys of methanotrophic taxa [14,15]. Up until very recently, the research supporting methanotrophy in the *Mycobacteriaceae* conflicted with numerous mechanistic studies focussed on the alkane and alkene degradation in strains such as *Mycobacterium chubuense* NBB4 and *Mycobacterium* sp. TY-6, where methane oxidation did not occur [16–19]. To the best of our knowledge, the recent cultivation of *Candidatus* Mycobacterium methanotrophicum from an acidic cave biofilm is the first to provide direct physiological evidence of aerobic methane oxidation via the sMMO complex in a member of the *Mycobacteriaceae* [20].

We note the recent discovery of *Candidatus* Mycobacterium methanotrophicum here because the phylogenetic analyses carried out as part of that study aligns with our own phylogenomic analyses identifying putative methanotrophs in the *Mycobacteriaceae*. In the original study detailing methanotrophy in *Candidatus* Mycobacterium methanotrophicum, the authors showed that multiple members of the *Mycobacteriaceae* including *Candidatus* Mycobacterium methanotrophicum carried *mmoX* and *mmoB* homologues [20]. We identified several of these strains in our phylogenomic analyses as members of the *Mycobacteriaceae* harbouring genes required for a complete or near-complete sMMO complex (e.g., *Mycobacterium rhodesia* A corresponding to *Mycolicibacterium rhodesia* NBB3, *Mycobacterium chubuense* A corresponding to *Mycolicibacterium chubuense* NBB4, *Mycobacterium moriokaense* corresponding to *Mycolicibacterium moriokaense*, *Mycobacterium elephantis* corresponding to *Mycolicibacterium elephantis*, and *Mycobacterium pulveris* corresponding to *Mycolicibacterium pulveris*) (Fig S12) [20].

We highlight these parallel observations to underscore how phylogenomic analyses can be used to challenge old metabolic paradigms. With several *Mycobacteriaceae* strains being available through culture collections, there is an opportunity to follow up on the recent evidence of methanotrophy in *Candidatus* Mycobacterium methanotrophicum to assess whether other members of the *Mycobacteriaceae* in different habitats have been overlooked with respect to their contributions to the global methane cycle.

Supplementary Note 4: Screening of predicted methanogen MAGs and the UBA148 family

Four MAGs from the unnamed family UBA148 were initially predicted to be methanogens. Upon curation, all MAGs from UBA148 were removed from the putative methanogen dataset because they lacked annotated *mcr* genes (see MAGs STB_81, STC_9, STD1_54, STD1_76, and STD2_203 in Supplementary Data 7 on the Open Science Framework (OSF) at DOI 10.17605/OSF.IO/6X5ZC). All other MAGs that were taxonomically classified to methanogen families but lacked the *mcrA* gene carried genes coding for subcomponent A2 of the methyl coenzyme M reductase and/or the *mcrC* and *mcrD* genes and were kept in the final dataset (see Supplementary Data 7 on the Open Science Framework (OSF) at DOI 10.17605/OSF.IO/6X5ZC).

According to GTDB release 89, UBA148 is in the order *Methanocellales*, which contains the methanogenic *Methanocellaceae* family [1,2]. Although MAGs belonging to the family UBA148 lacked *mcr* genes, they displayed >75 % completion of the hydrogenotrophic methanogenesis pathways alongside complete CODH/ACS complexes, and in some cases, the ability to convert acetate to acetyl-CoA (see Supplementary Data 1 and 7 on the Open Science Framework (OSF) at DOI 10.17605/OSF.IO/6X5ZC). We also observed that the *mtrA* and *mtrH* genes from the *mtrEDCBAGH* gene cluster coding for the tetrahydromethanopterin S-methyltransferase, which produces methyl coenzyme M [3,4], were present in UBA148 MAGs (see MAGs STB_81, STC_9, STD1_54, STD1_76, and STD2_203 in Supplementary Data 7 on the Open Science Framework (OSF) at DOI 10.17605/OSF.IO/6X5ZC).

In many instances, the abundance of UBA148 MAGs was higher than putative methanogens, particularly in older MSW (see Supplementary Data 1 on the Open Science Framework (OSF) at DOI 10.17605/OSF.IO/6X5ZC). We speculate that this may be tied to the

confirmed or suspected oxygen infiltration in older landfill cells (i.e., A, B, and C). In manually verifying annotations for UBA148 MAGs, we observed that genes coding antioxidant enzymes including superoxide reductase, peroxiredoxin, and rubrerythrin, were present alongside genes associated with redox buffer systems such as thioredoxin and rubredoxin (see MAGs STB81, STC_9, STD1_54, STD1_76 in Supplementary Data 7 on the Open Science Framework (OSF) at DOI 10.17605/OSF.IO/6X5ZC). The presence of similar genes has been noted as potential adaptations to tolerating oxidative stress in bona fide methanogens [5].

The major difference between members of family UBA148 and methanogenic lineages in this study is that UBA148 genomes lacked genes coding for the *mcr* complex, which houses an extremely oxygen sensitive Ni(I) cofactor [6,7]. We speculate that members of the UBA148 family may gain a competitive advantage through the elimination of the oxygen sensitive *mcr* complex to support an autotrophic lifestyle in habitats where oxygen infiltration would directly inhibit *mcr*-dependent methanogenesis. This hypothesis has yet to be tested but underscores the need to understand how methanogens tolerate oxidative stress in ageing landfills and how these adaptations control long term methane production.

Supplementary Figures

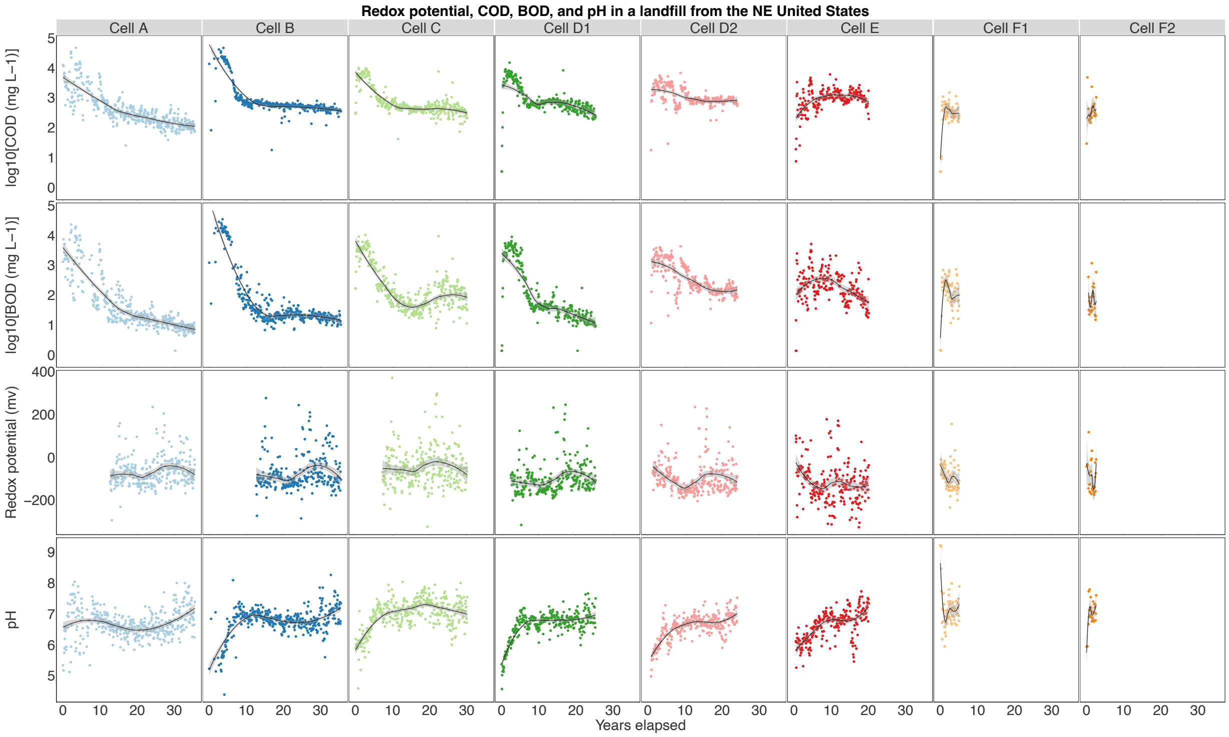

Figure S1: Aqueous geochemistry of landfill leachate collected from landfill cells A, B, C, D1, D2, E, F1 and F2 from 1983 to 2019. The number of observations for chemical oxygen demand (COD) at each landfill cell are n = 404 for cell A, n = 388 for cell B, n = 361 for cell C, n = 305 for cell D1, n = 278 for cell D2, n = 237 for cell E, n = 61 for cell F1, and n = 28 for cell F2. The number of observations for biological oxygen demand (BOD) at each landfill cell are n = 386 for cell A, n = 370 for cell B, n = 351 for cell C, n = 295 for cell D1, n = 268 for cell D2, n = 227 for cell E, n = 61 for cell F1, n = 26 for cell F2. The number of observations for redox potential at each landfill cell are n = 275 for cell A, n = 275 for cell B, n = 275 for cell C, n = 275 for cell D1, n = 274 for cell D2, n = 237 for cell E, n = 61 for cell F, and n = 28 for cell F2. The number of observations for pH at each landfill cell are n = 404 for cell A, n = 388 for cell B, n = 360 for cell C, n = 305 for cell D1, n = 278 for cell D2, n = 237 for cell E, n = 61 for cell F1, and n = 28 for cell F2. Data was compiled from monitoring records provided by the site management. Loess curves are presented to smooth temporal trends. Source Data are provided as a Source Data file.

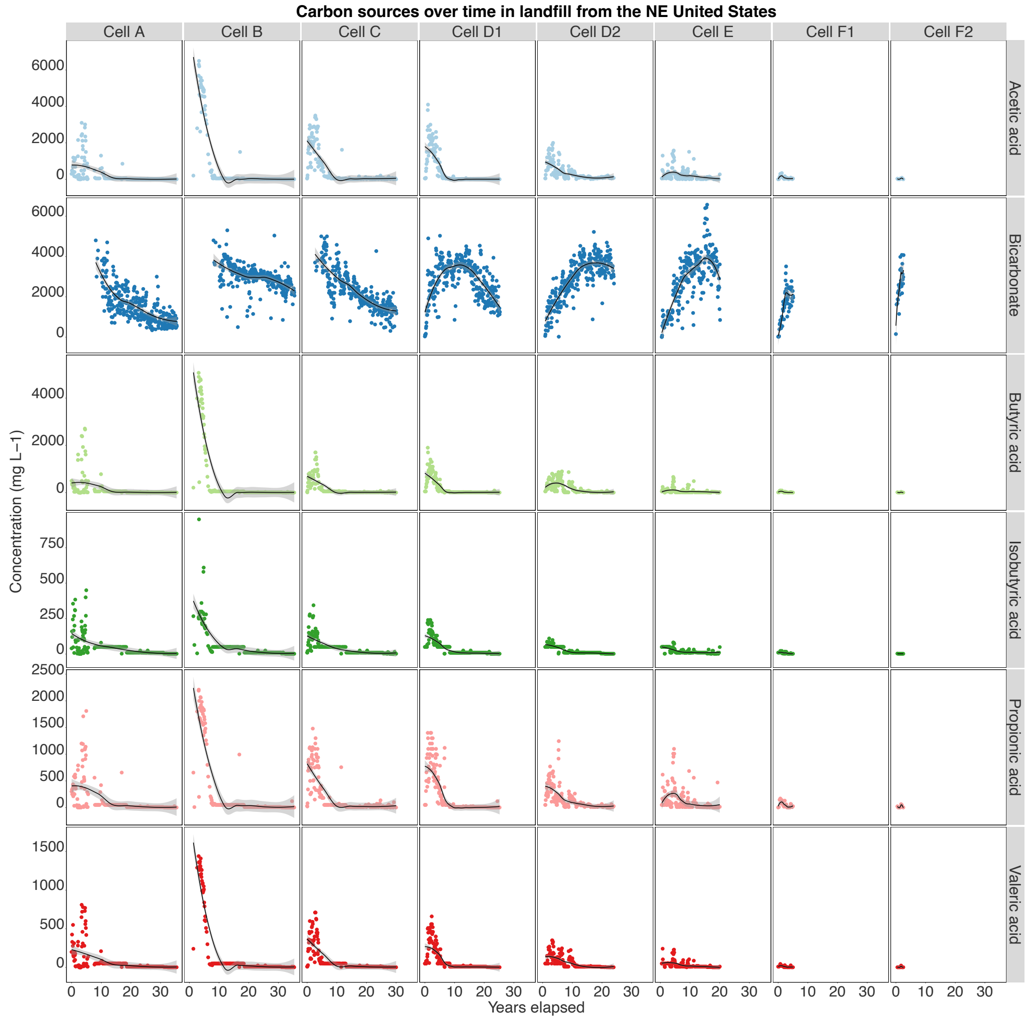

Figure S2: Organic acid and bicarbonate concentrations in landfill leachate collected from landfill cells A, B, C, D1, D2, E, F1 and F2 from 1983 to 2019. The number of observations for acetic acid at each landfill cell are n = 169 for cell A, n = 152 for cell B, n = 160 for cell C, n = 227 for cell D1, n = 200 for cell D2, n = 159 for cell E, n = 21 for cell F1, and n = 9 for cell F2. The number of observations for bicarbonate at each landfill cell are n = 309 for cell A, n = 309 for cell B, n = 309 for cell C, n = 305 for cell D1, n = 278 for cell D2, n = 236 for cell E, n = 61 for cell F1, and n = 28 for cell F2. The number of observations for butyric acid at each landfill cell are n = 171 for cell A, n = 152 for cell B, n = 160 for cell C, n = 227 for cell D1, n = 200 for cell D2, n = 159 for cell E, n = 21 for cell F1, and n = 9 for cell F2. The number of observations for isobutyric acid at each landfill cell are n = 172 for cell A, n = 153 for cell B, n = 160 for cell C, n = 226 for cell D1, n = 199 for cell D2, n = 158 for cell E, n = 21 for cell F1, and n = 9 for cell F2. The number of observations for propionic acid at each landfill cell are n = 172 for cell A, n = 153 for cell B, n = 160 for cell C, n = 227 for cell D1, n = 200 for cell D2, n = 159 for cell E, n = 21 for cell F1, n = 9 for cell F2. The number of observations for valeric acid at each landfill cell are n = 171 for cell A, n = 152 for cell B, n = 160 for cell C, n = 226 for cell D1, n = 199 for cell D2, n = 158 for cell E, n = 21 for cell F1, n = 9 for cell F2. These data were compiled from monitoring records provided by the site management. Loess curves have been fitted to the data to visualize smoothed temporal trends. Source Data are provided as a Source Data file.

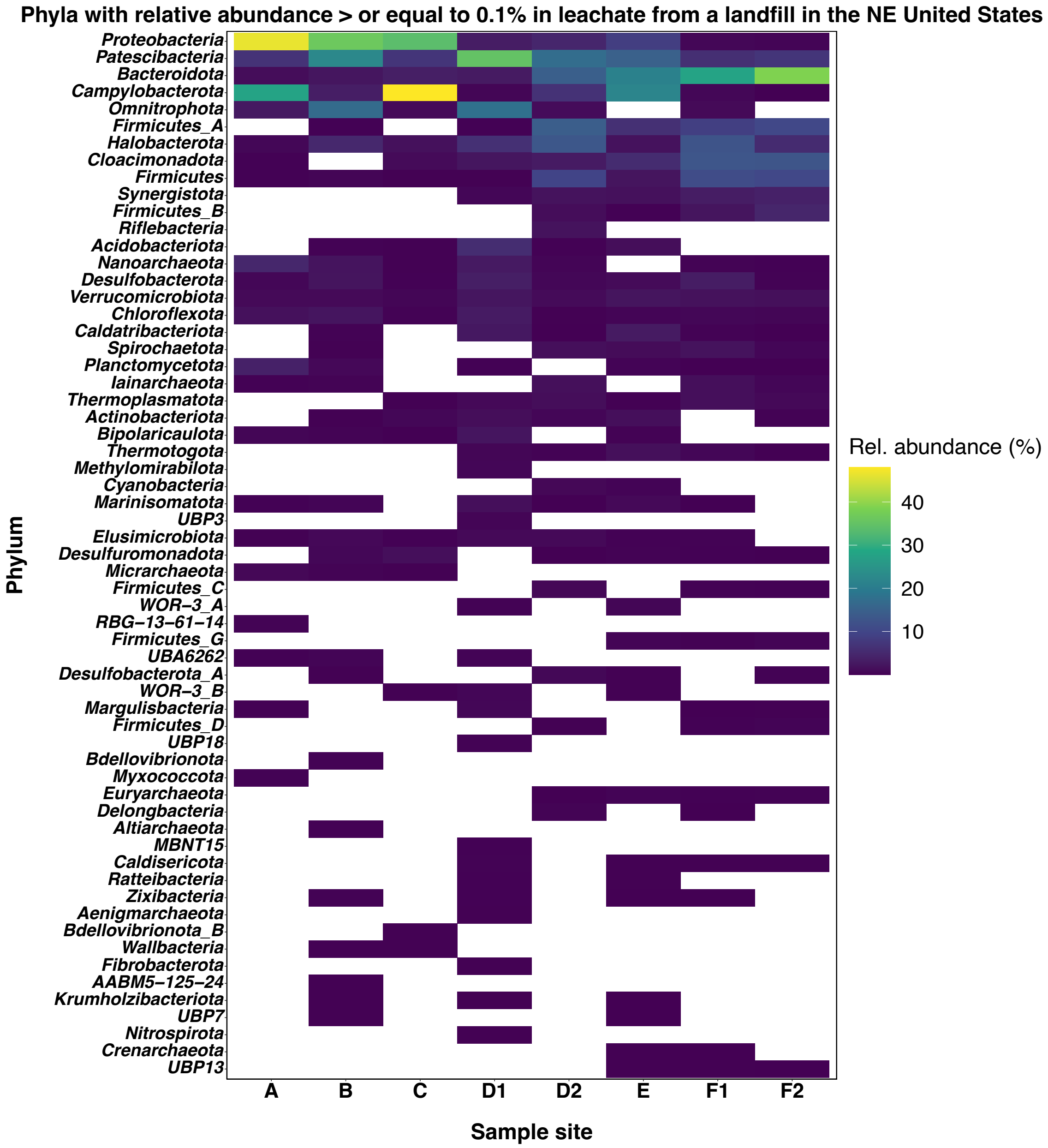

Figure S3: Heatmap showing all phyla with ≥0.1% relative abundance (rel. abundance) detected in any one landfill leachate sample. Relative abundance was calculated based on the mean coverage of all scaffolds within a genome bin to obtain a mean coverage value for each metagenome-assembled genome (MAG). Afterwards, the individual MAG coverage values were summed for each site to obtain the denominator to calculate relative abundance. Each individual MAG coverage value was divided by that denominator and multiplied by 100 to obtain a relative abundance that was summed at the phylum level. Source Data are provided as a Source Data file.

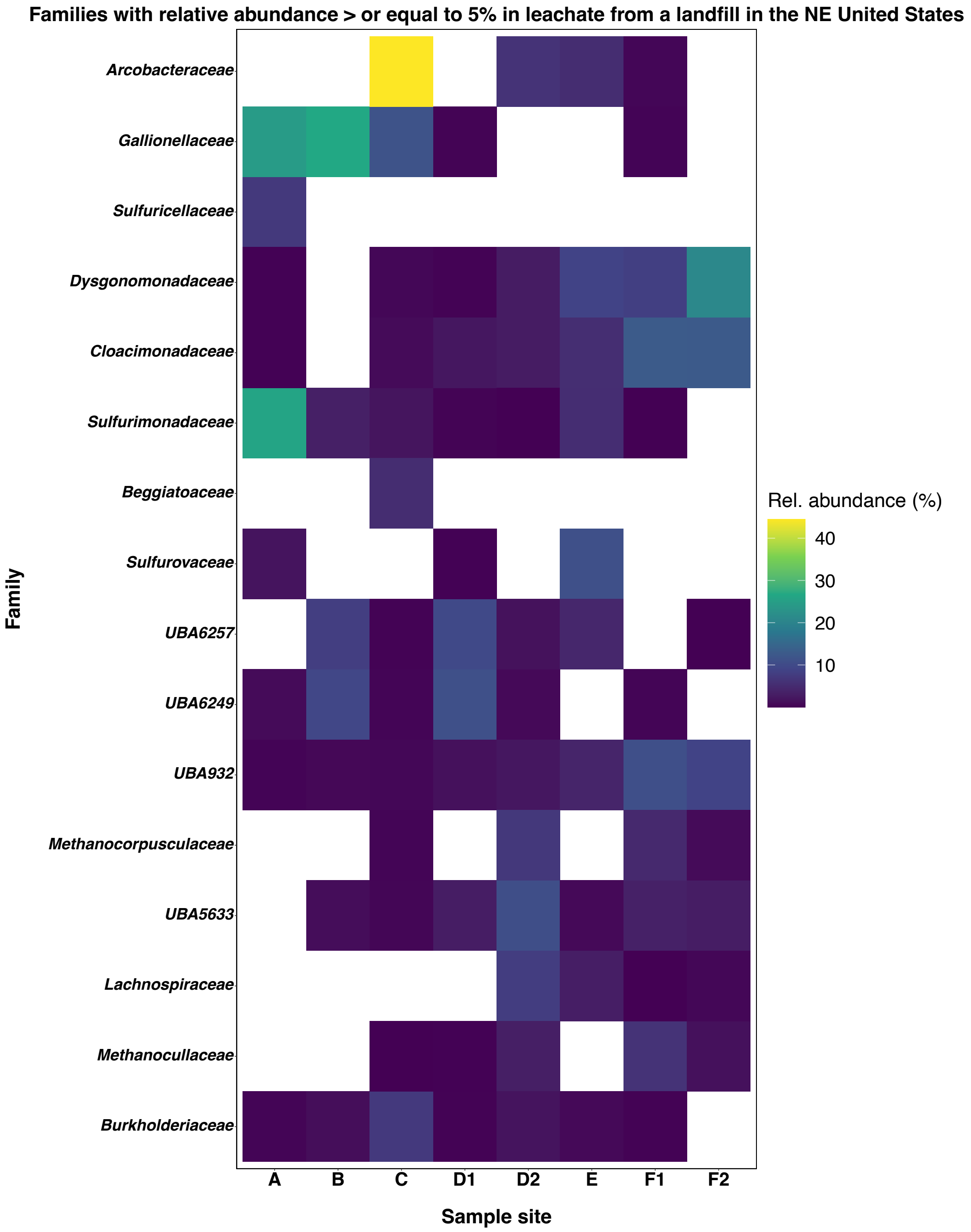

Figure S4: Heatmap showing all families with ≥5% relative abundance (rel. abundance) detected in any one landfill leachate sample. Relative abundance was calculated based on the mean coverage of all scaffolds within a genome bin to obtain a mean coverage value for each metagenome-assembled genome (MAG). Afterwards, the individual MAG coverage values were summed for each site to obtain the denominator to calculate relative abundance. Each individual MAG coverage value was divided by that denominator and multiplied by 100 to obtain a relative abundance that was summed at the family level. Source Data are provided as a Source Data file.

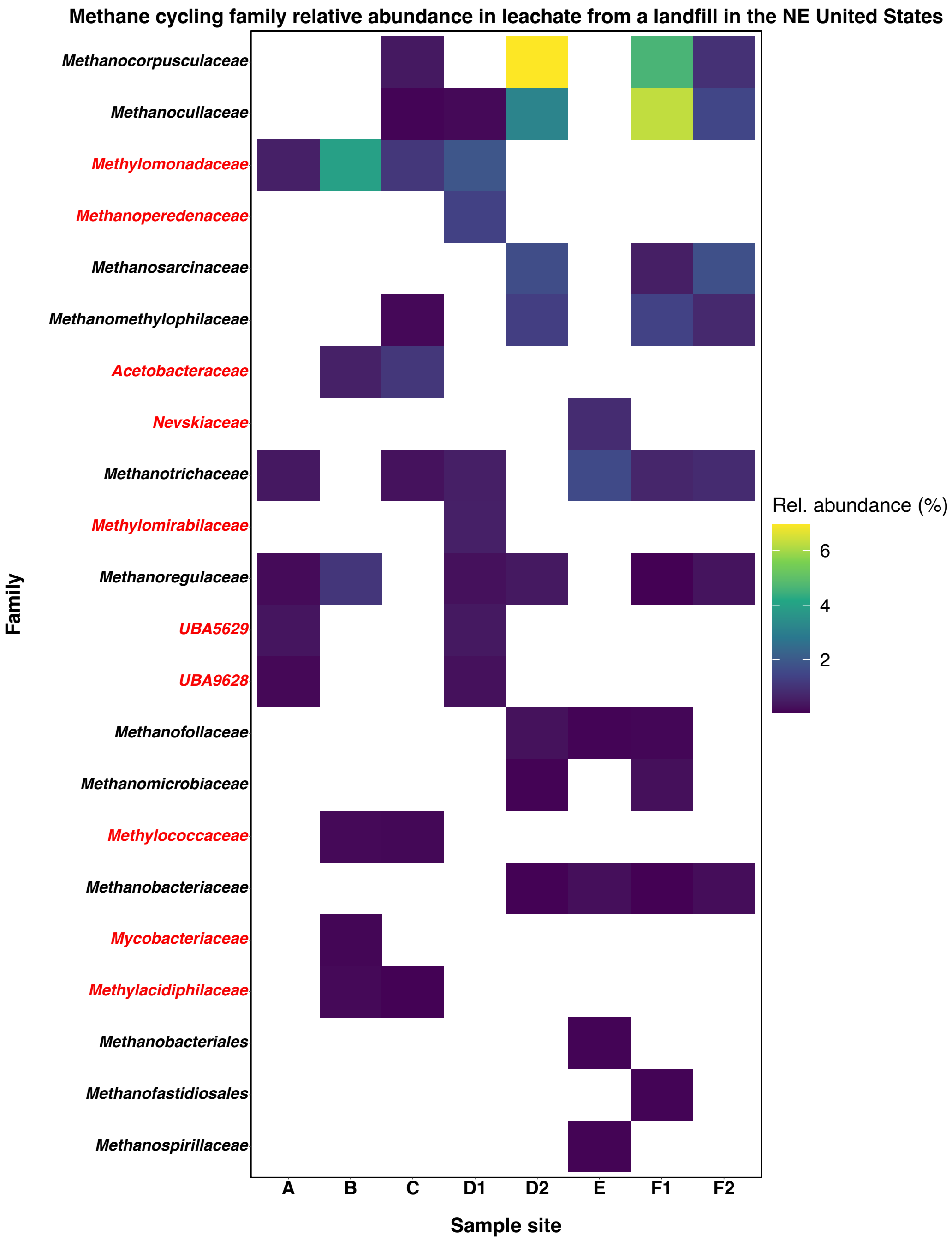

Figure S5: Relative abundance (rel. abundance) of putative methanogenic (black) and methanotrophic (red) metagenome-assembled genomes (MAG) summarized at the family level. The heatmap colour gradient has been scaled to the most abundant putative methanogenic MAG. Relative abundance was calculated based on the mean coverage of all scaffolds within a genome bin to obtain a mean coverage value for MAG. Afterwards, the individual MAG coverage values were summed for each site to obtain the denominator to calculate relative abundance. Each individual MAG coverage value was divided by that denominator and multiplied by 100 to obtain a relative abundance that was summed at the family level. Source Data are provided as a Source Data file.

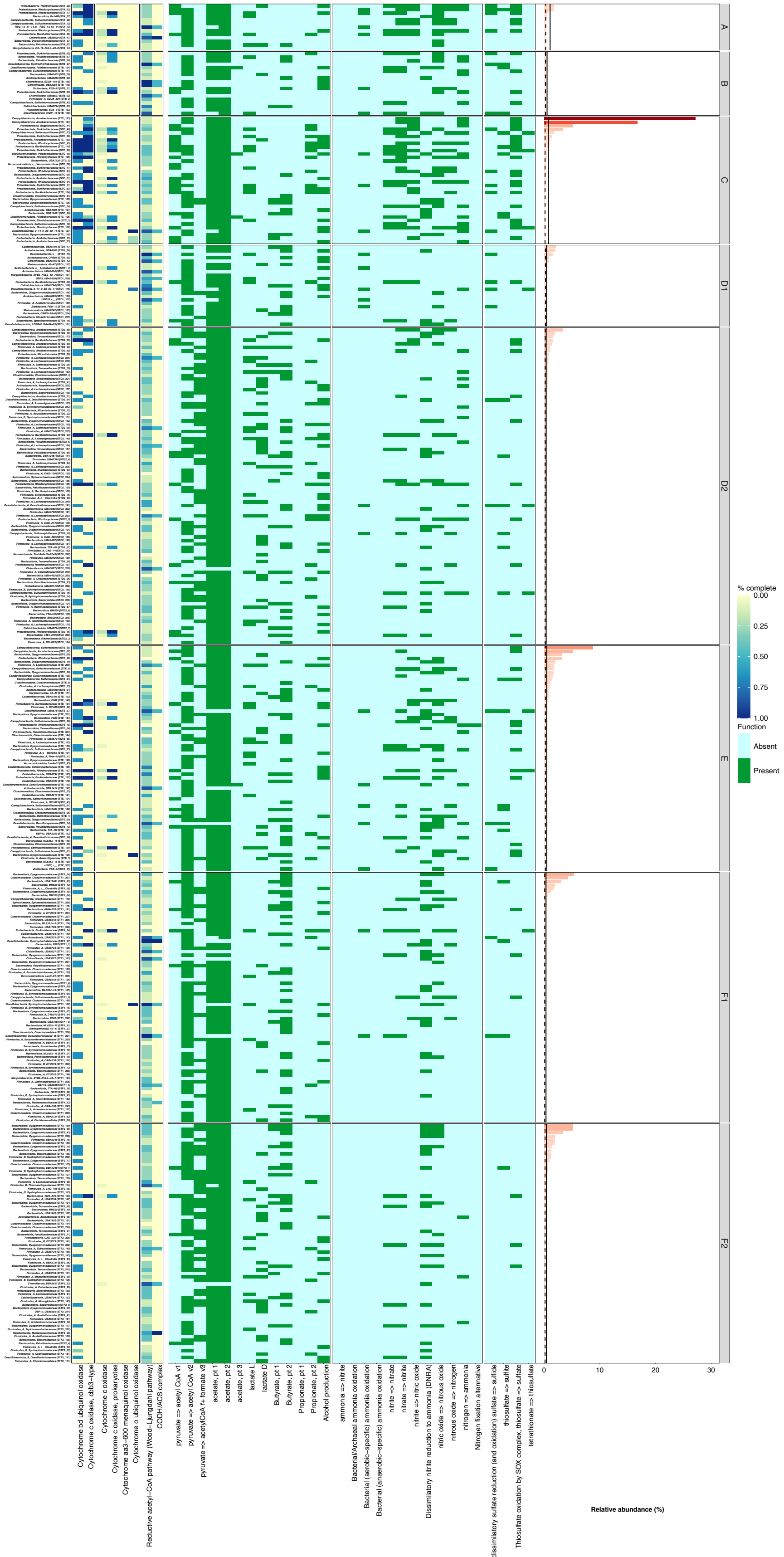

Figure S6: Metabolic heatmap and relative abundance data for putative acetogenic metagenome-assembled genomes (MAGs). The abbreviation CODH/ACS denotes the completion of the carbonic anhydrase/acetyl-CoA synthase pathway. Relative abundance values have been scaled to the most abundant MAG in the entire data set. Solid black vertical lines denote the mean relative abundance calculated for MAGs at the whole-community level and dashed black lines denote the median relative abundance calculated for MAGs at the whole-community level. Source Data are provided as a Source Data file.

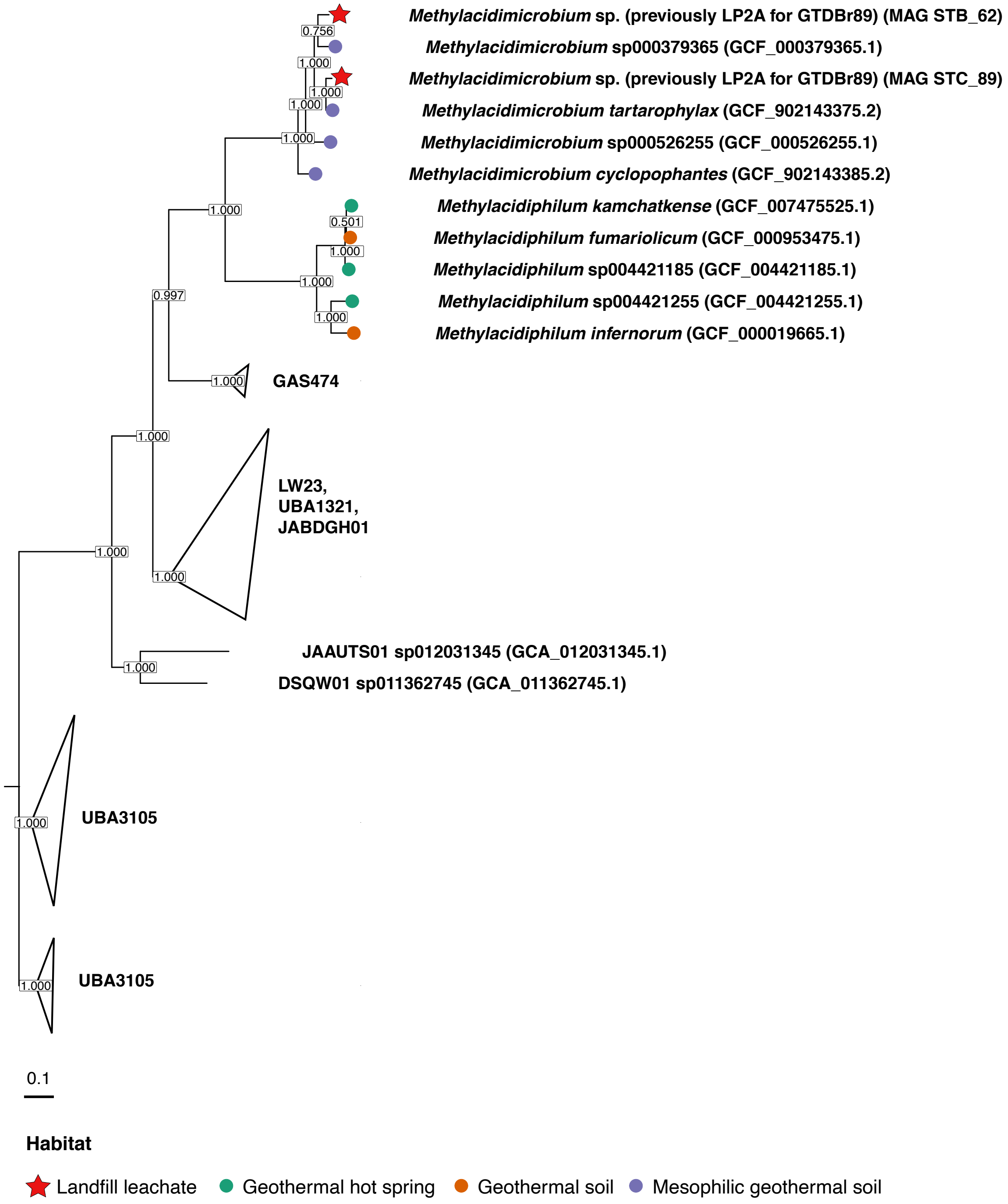

Figure S7: Rooted phylogenetic for *Methylacidiphilaceae* genomes retrieved from the landfill (denoted by a red star) and Genome Taxonomy Database (GTDB) generated by GToTree. The unnamed family UBA3105 within the order *Methylacidiphilales* was used as the outgroup. Coloured circles correspond to the different environmental sources associated with the NCBI biosample entries for each genome. The scale bar denotes amino acid substitutions per site. Source Data are provided as a Source Data file.

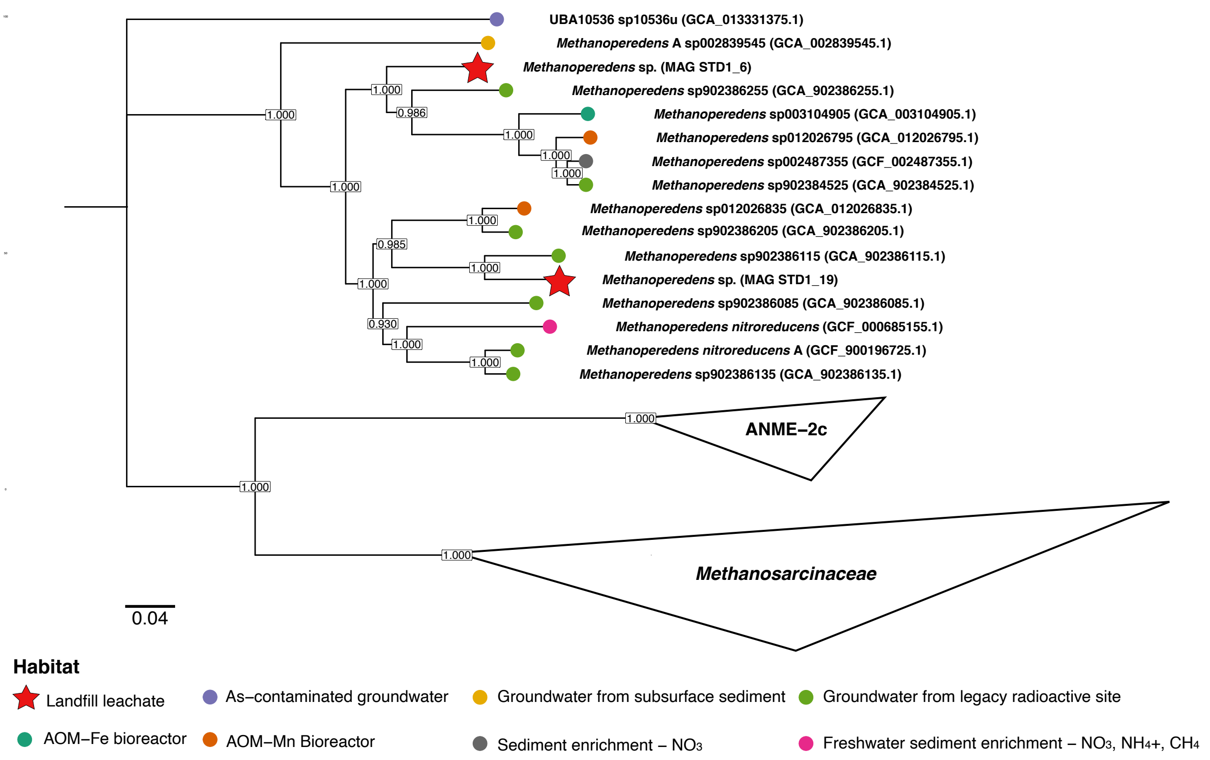

Figure S8: Rooted phylogenetic tree for *Methanoperedenaceae* genomes retrieved from the landfill (denoted by a red star) and Genome Taxonomy Database (GTDB) generated by GToTree. The families ANME-2c and *Methanosarcinaceae* were used as outgroups. Coloured circles correspond to the different environmental sources associated with the NCBI biosample entries for each genome. The scale bar denotes amino acid substitutions per site. Source Data are provided as a Source Data file.

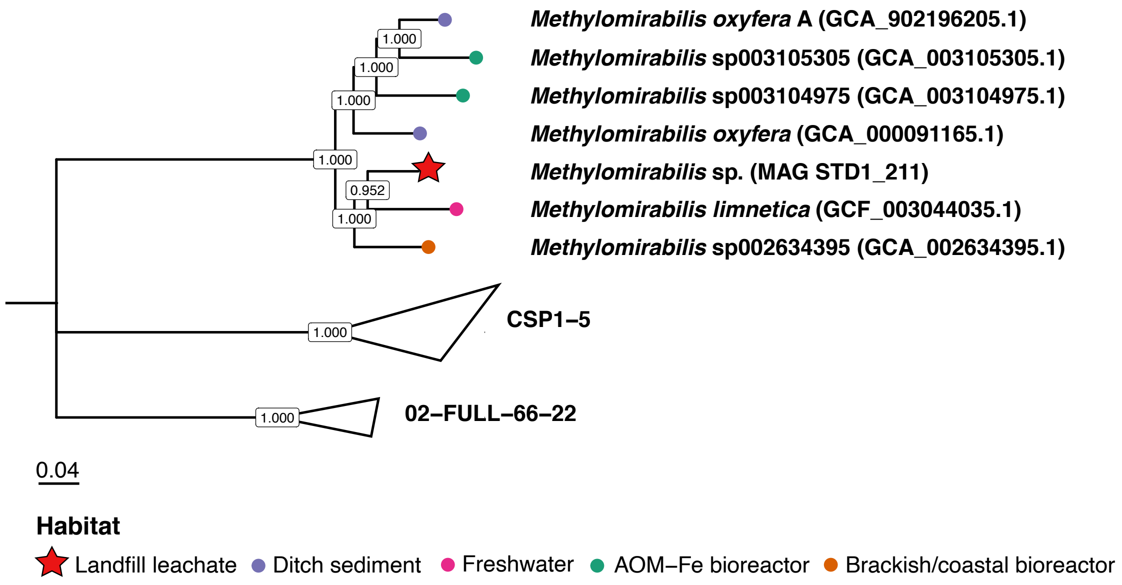

Figure S9: Rooted phylogenetic tree for *Methylomirabilaceae* genomes retrieved from the landfill (denoted by a red star) and Genome Taxonomy Database (GTDB) generated by GToTree. The unnamed families CSP1-5 and 02-FULL-66-22 were used as outgroups. Coloured circles correspond to the different environmental sources associated with the NCBI biosample entries for each genome. The abbreviation AOM-Fe denotes anaerobic oxidation of methane in the presence of iron. The scale bar denotes amino acid substitutions per site. Source Data are provided as a Source Data file.

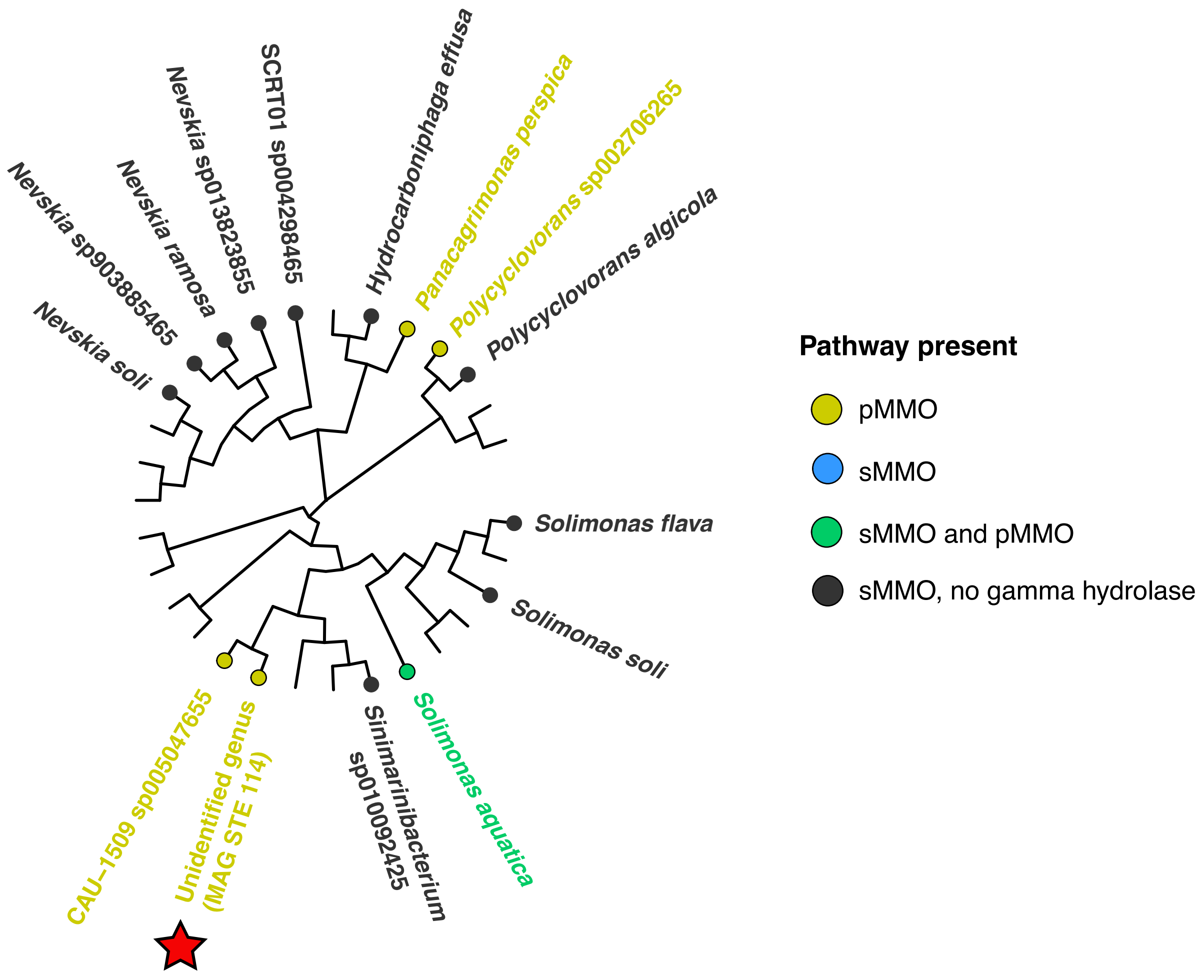

Figure S10: Unrooted phylogenetic tree for 30 *Nevskiaceae* genomes retrieved from the landfill (shown with a red star) and Genome Taxonomy Database (GTDB), representing all currently available *Nevskiaceae* genomes. Coloured circles and text denote the different types of putative methanotrophs identified by including the Pfam entries for the pMMO and sMMO pathways during the tree building process in GToTree. Source Data are provided as a Source Data file.

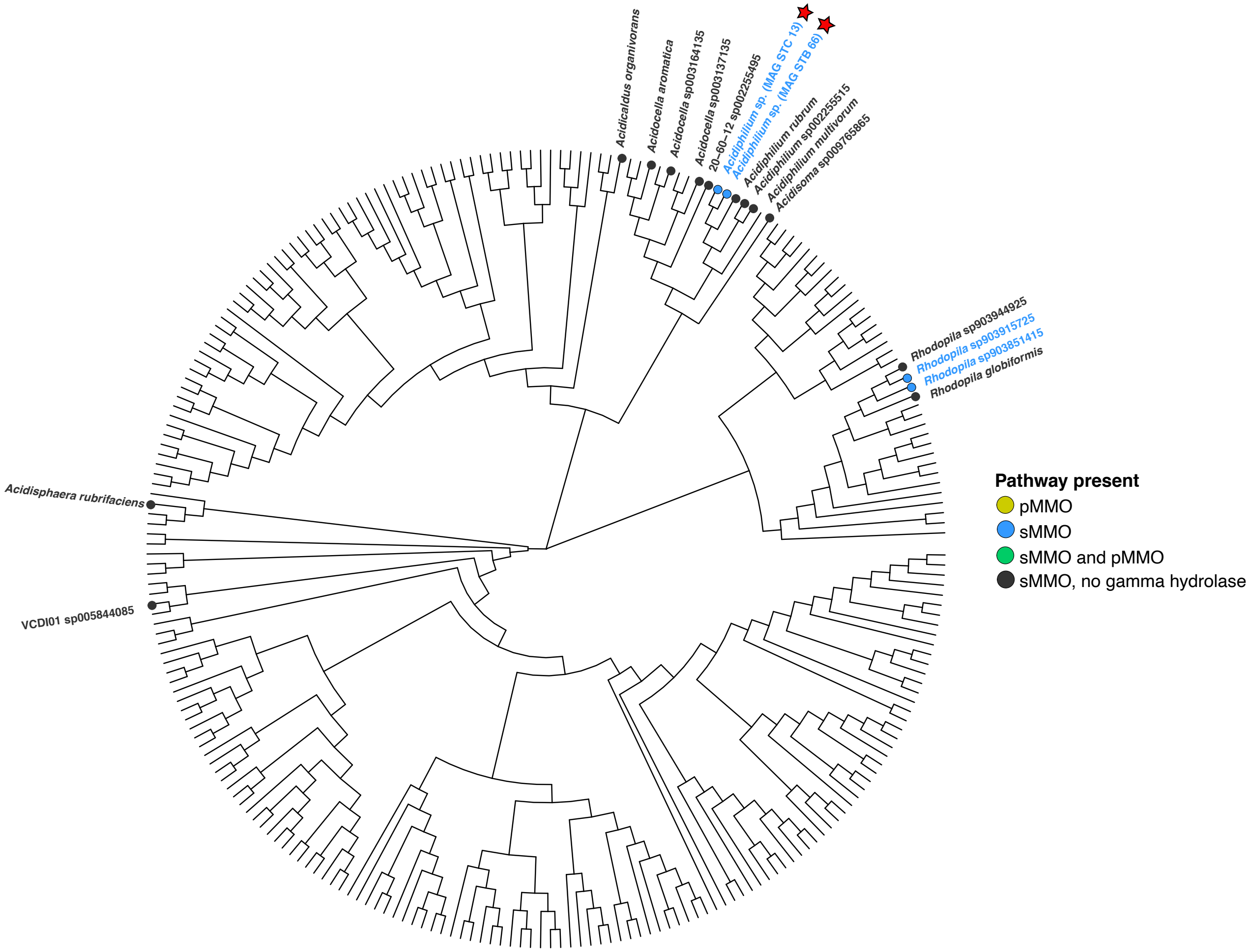

Figure S11: Unrooted phylogenetic tree for 254 *Acetobacteraceae* genomes retrieved from the landfill (shown with a red star) and Genome Taxonomy Database (GTDB), representing all currently available *Acetobacteraceae* genomes. Coloured circles and text denote the different types of putative methanotrophs identified by including the Pfam entries for the pMMO and sMMO pathways during the tree building process in GToTree. Source Data are provided as a Source Data file.

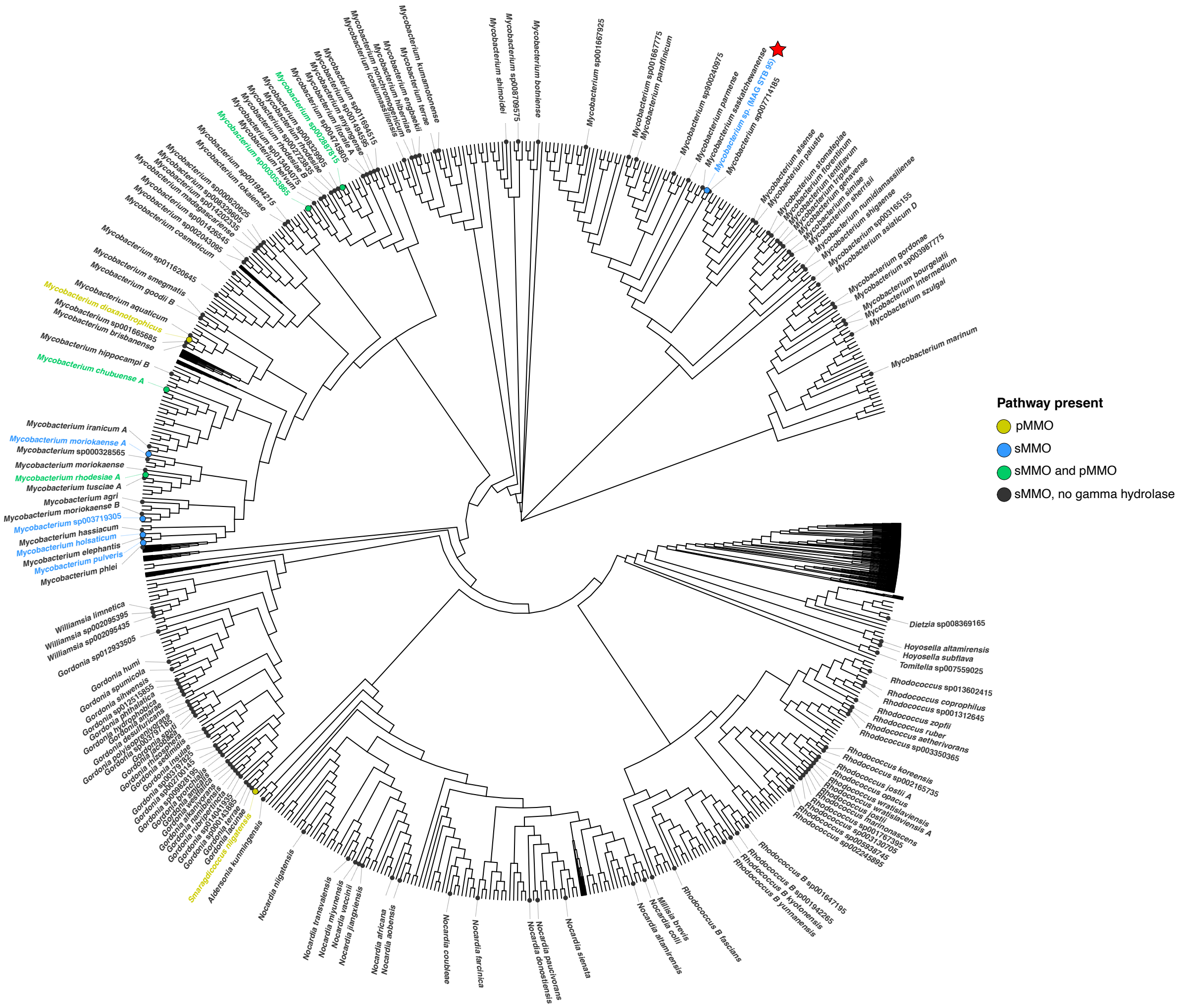

Figure S12: Unrooted phylogenetic tree for *Mycobacteriaceae* genomes retrieved from the landfill (shown with a red star) and Genome Taxonomy Database (GTDB). Coloured circles and text denote the different types of putative methanotrophs identified by including the Pfam entries for the pMMO and sMMO pathways during the tree building process in GToTree. This tree includes all 811 representative genomes available through GTDB. Due to the size of the tree, clades with no hits for the categories of methane oxidation pathways present were scaled down to 10% of their size and hits for the pMMO, sMMO, and sMMO and pMMO categories had their symbols increased in size. Source Data are provided as a Source Data file.

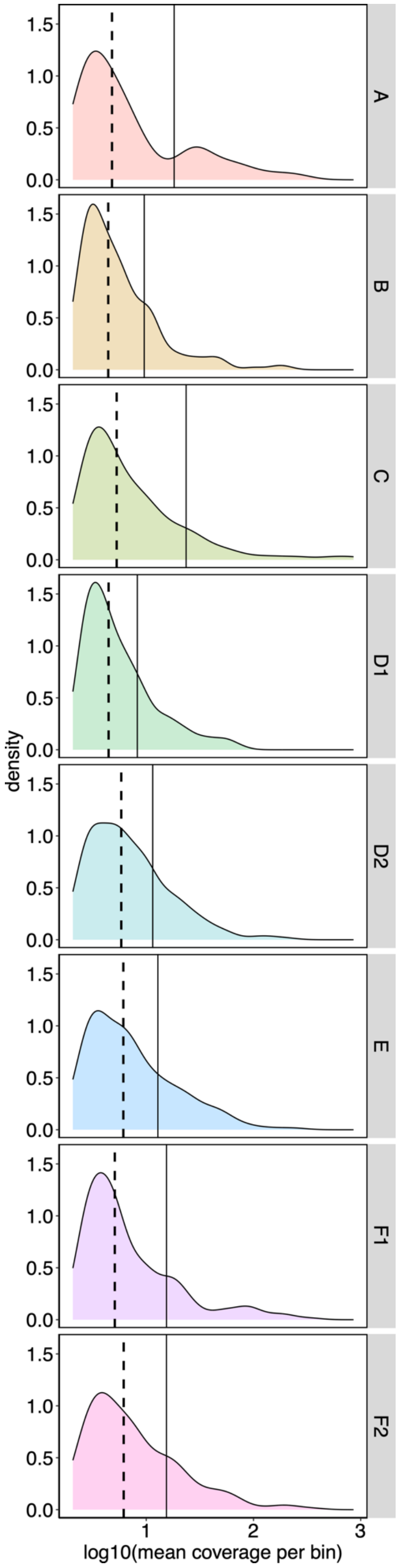

Figure S13: Density plots showing the distribution of mean coverage values calculated for all metagenome-assembled genomes (MAGs) recovered from landfill cells A, B, C, D1, D2, E, F1, and F2. Solid black vertical lines denote the mean coverage calculated for MAGs at the whole-community level and dashed black lines denote the median coverage calculated for MAGs at the whole-community level. Source Data are provided as a Source Data file.

Table S1: Landfill site characteristics and geochemistry on sampling day 2019-02-12. Source Data are provided as a Source Data file. The abbreviation COD denotes chemical oxygen demand, the abbreviation BOD denotes biological oxygen demand.

Landfill cell	Size (Acres)	Beginning of filling	End of filing	Tons landfilled (T)	Leachate generated (Gallons)	Leachate directly recirculated (Gallons)	COD	BOD	BOD/COD	pH	Redox potential (mV)
A	8.9	September 1980	September 1982	136,314	58,048,161	2,656,153	107.00	10.5	0.098	7.55	-11.0
B	18.3	September 1982	October 1988	395,790	125,564,005	11,957,829	518.00	15.0	0.029	7.5	-81.0
C	19.4	October 1988	December 1993	630,966	47,269,855	4,133,288	310.00	45.6	0.15	7.7	-42.0
D1	22.5	October 1993	October 1998	595,082	52,975,487	4,076,454	347.00	13.7	0.039	7.66	-84.0
D2[a]	NA	NA	NA	NA	NA	NA	784.00	125.0	0.16	7.68	-65.0
C/D valley	7	July 1998	July 1999	143,401	NA	2,086,350	NA	NA	NA	NA	NA
E	32.5	June 1999	October 2014	2,395,873	64,241,861	4,450,465	286.00	36.1	0.13	7.50	-14.0
F1[b]	25.6	December 2014	Present	770,171	20,928,440	NA	378.00	63.7	0.17	8.05	-85.0
F2	14.8	June 2016	Present	NA	NA	NA	1520.00	859.0	0.56	7.760	13.3

a- Values were provided by site owners for cell D, not specifically D1 or D2. Values for cell D are displayed in the row for D1.
b- Values for tons landfilled and leachate generated are provided for landfill cell F overall.

Table S2: Summary statistics produced for all landfill metagenomes using BBMap (https://sourceforge.net/projects/bbmap/). Source Data are provided as a Source Data file. The abbreviation MAG denotes metagenome-assembled genome. The abbreviation Avg denotes average and the abbreviation STDEV denotes standard deviation.

Site	# Gbp sequenced	# of scaffolds	# of contigs	Total scaffold length (bp)	Total contig length (bp)	Gap %	N50 N90 for scaffolds	L50 L90 for scaffolds	N50 N90 for contigs	L50 L90 for contigs	Maximum scaffold length (bp)	Maximum contig length (bp)	Sequence count for scaffolds >50 KB	Sequence size % for scaffolds >50 KB	Avg GC content	STDEV for GC content	Gbp reads in MAGs	Min. cov. for single MAG	Max. cov for single MAG	Mean cov for all MAGs	Median cov for all MAGs
A	33.07	1.05E+06	1.06E+06	2.19E+09	2.19E+09	0.013	2.22E+05<br>8.38E+05	2.06E+03<br>1.10E+03	2.26E+05<br>8.44E+05	2.04E+03<br>1.10E+03	1.55E+06	1.55E+06	1.30E+03	6.65	0.50	0.11232	7.79	2.07	267.78	18.21	4.79
B	29.23	1.18E+06	1.19E+06	2.56E+09	2.56E+09	0.014	2.42E+05<br>7.99E+05	2.20E+03<br>1.21E+03	2.46E+05<br>8.06E+05	2.19E+03<br>1.20E+03	1.57E+06	1.57E+06	1.40E+03	6.36	0.47	0.10202	7.59	2.14	195.25	9.58	4.42
C	34.48	9.28E+05	9.41E+05	2.13E+09	2.13E+09	0.016	1.76E+05<br>7.27E+05	2.43E+03<br>1.13E+03	1.80E+05<br>7.36E+05	2.40E+03<br>1.13E+03	1.32E+06	1.10E+06	1.38E+03	7.29	0.48	0.12012	17.43	2.30	857.19	23.54	5.30
D1	32.25	1.52E+06	1.53E+06	3.17E+09	3.16E+09	0.013	3.40E+05<br>6.65E+05	2.08E+03<br>1.49E+03	3.44E+05<br>6.72E+05	2.07E+03<br>1.48E+03	1.17E+06	1.17E+06	1.32E+03	4.58	0.46	0.10489	6.74	2.16	76.15	8.25	4.45
D2	29.60	1.07E+06	1.08E+06	2.73E+09	2.73E+09	0.022	1.74E+05<br>5.74E+05	2.94E+03<br>1.44E+03	1.79E+05<br>5.83E+05	2.90E+03<br>1.44E+03	7.84E+05	5.33E+05	2.31E+03	7.95	0.43	0.10683	12.54	2.24	205.95	11.49	5.85
E	22.89	8.14E+05	8.21E+05	1.95E+09	1.95E+09	0.02	1.49E+05<br>6.33E+05	2.61E+03<br>1.15E+03	1.52E+05<br>6.38E+05	2.59E+03<br>1.15E+03	7.14E+05	6.63E+05	1.16E+03	5.56	0.46	0.11564	10.45	2.26	236.45	12.84	6.11
F1	35.13	1.06E+06	1.07E+06	2.51E+09	2.51E+09	0.022	2.02E+05<br>7.32E+05	2.60E+03<br>1.23E+03	2.07E+05<br>7.39E+05	2.57E+03<br>1.23E+03	1.24E+06	1.10E+06	1.29E+03	4.70	0.45	0.10868	18.67	2.08	353.67	15.44	5.09
F2	30.67	9.50E+05	9.61E+05	2.29E+09	2.29E+09	0.021	1.77E+05<br>7.37E+05	2.66E+03<br>1.16E+03	1.81E+05<br>7.45E+05	2.63E+03<br>1.15E+03	7.35E+05	7.35E+05	1.30E+03	4.93	0.43	0.09613	16.18	2.12	347.40	15.43	6.16

Supplementary References

1. Chaumeil, P. A., Mussig, A. J., Hugenholtz, P. & Parks, D. H. GTDB-Tk: a toolkit to classify genomes with the Genome Taxonomy Database. *Bioinformatics* (2019) doi:10.1093/bioinformatics/btz848.
2. Parks, D. H. *et al.* GTDB: an ongoing census of bacterial and archaeal diversity through a phylogenetically consistent, rank normalized and complete genome-based taxonomy. *Nucleic Acids Res.* **50**, D785–D794 (2022).
3. Hippler, B. & Thauer, R. K. The energy conserving methyltetrahydromethanopterin:coenzyme M methyltransferase complex from methanogenic archaea: Function of the subunit MtrH. *FEBS Lett.* **449**, 165–168 (1999).
4. Harms, U., Weiss, D. S., Gärtner, P., Linder, D. & Thauer, R. K. The energy conserving N5-methyltetrahydromethanopterin:coenzyme M methyltransferase complex from *Methanobacterium thermoautotrophicum* is composed of eight different subunits. *Eur. J. Biochem.* **228**, 640–648 (1995).
5. Lyu, Z. & Lu, Y. Metabolic shift at the class level sheds light on adaptation of methanogens to oxidative environments. *ISME J.* **12**, 411–423 (2018).
6. Goubeaud, M., Schreiner, G. & Thauer, R. K. Purified methyl-coenzyme-M deductase is activated when the enzyme-bound coenzyme F430 is reduced to the nickel(I) oxidation state by titanium(III) citrate. *Eur. J. Biochem.* **243**, 110–114 (1997).
7. Duin, E. C., Cosper, N. J., Mahlert, F., Thauer, R. K. & Scott, R. A. Coordination and geometry of the nickel atom in active methyl-coenzyme M reductase from *Methanothermobacter marburgensis* as detected by X-ray absorption spectroscopy. *JBIC J. Biol. Inorg. Chem.* **8**, 141–148 (2003).

8. Nobu, M. K., Narihiro, T., Kuroda, K., Mei, R. & Liu, W.-T. Chasing the elusive Euryarchaeota class WSA2: Genomes reveal a uniquely fastidious methyl-reducing methanogen. *ISME J.* **10**, 2478–2487 (2016).

9. Harms, U. & Thauer, R. K. Methylcobalamin:coenzyme M methyltransferase isoenzymes MtaA and MtbA from *Methanosarcina barkeri*: Cloning, sequencing and differential transcription of the encoding genes, and functional overexpression of the *mtaA* gene in *Escherichia coli*. *Eur. J. Biochem.* **235**, 653–659 (1996).

10. Tallant, T. C., Paul, L. & Krzycki, J. A. The MtsA subunit of the methylthiol:coenzyme M methyltransferase of *Methanosarcina barkeri* catalyses both half-reactions of corrinoid-dependent dimethylsulfide: coenzyme M methyl transfer. *J. Biol. Chem.* **276**, 4485–4493 (2001).

11. Nechaeva, N. B. Two species of methane oxidizing mycobacteria. *Mikrobiologiya* vol. 18 310–317 (1949).

12. Reed, W. M. & Dugan, P. R. Isolation and characterization of the facultative methylotroph *Mycobacterium* ID-Y. *J. Gen. Microbiol.* **133**, 1389–1395 (1987).

13. Smit, N. T. *et al.* Novel hydrocarbon-utilizing soil mycobacteria synthesize unique mycocerosic acids at a Sicilian everlasting fire. *Biogeosciences* **18**, 1463–1479 (2021).

14. Dedysh, S. N. & Knief, C. Diversity and phylogeny of described aerobic methanotrophs. in *Methane Biocatalysis: Paving the Way to Sustainability* 17–42 (Springer International Publishing, 2018). doi:10.1007/978-3-319-74866-5_2.

15. Smith, G. J. & Wrighton, K. C. Metagenomic approaches unearth methanotroph phylogenetic and metabolic Diversity. *Curr. Issues Mol. Biol.* 57–84 (2019) doi:10.21775/cimb.033.057.

16. Martin, K. E., Ozsvar, J. & Coleman, N. V. SmoXYB1C1Z of *Mycobacterium* sp strain NBB4: a soluble methane monooxygenase (sMMO)-like enzyme, active on C-2 to C-4 alkanes and alkenes. *Appl. Environ. Microbiol.* **80**, 5801–5806 (2014).

17. Coleman, N. V *et al.* Hydrocarbon monooxygenase in *Mycobacterium*: recombinant expression of a member of the ammonia monooxygenase superfamily. *ISME J.* **6**, 171–182 (2012).

18. Coleman, N. V *et al.* Untangling the multiple monooxygenases of *Mycobacterium chubuense* strain NBB4, a versatile hydrocarbon degrader. *Environ. Microbiol. Rep.* **3**, 297–307 (2011).

19. Kotani, T., Kawashima, Y., Yurimoto, H., Kato, N. & Sakai, Y. Gene structure and regulation of alkane monooxygenases in propane-utilizing *Mycobacterium* sp. TY-6 and *Pseudonocardia* sp. TY-7. *J. Biosci. Bioeng.* **102**, 184–192 (2006).

20. Van Spanning, R. J. M. *et al.* Methanotrophy by a *Mycobacterium* species that dominates a cave microbial ecosystem. *Nat. Microbiol.* **7**, 2089–2100 (2022).
