# Supplementary material for: Microbial methane cycling in a landfill on a decadal time scale
Source: Nat Commun. 2023 Nov 16;14:7402. doi: 10.1038/s41467-023-43129-x (PMC10654671; doi:10.1038/s41467-023-43129-x)
Supplement: Supplementary file 2 — Reporting Summary [file 41467_2023_43129_MOESM2_ESM.pdf]

nature portfolio

Corresponding author(s): Daniel Grégoire and Laura Hug

Last updated by author(s): Oct 5, 2023

Reporting Summary

Nature Portfolio wishes to improve the reproducibility of the work that we publish. This form provides structure for consistency and transparency in reporting. For further information on Nature Portfolio policies, see our Editorial Policies and the Editorial Policy Checklist.

Statistics

For all statistical analyses, confirm that the following items are present in the figure legend, table legend, main text, or Methods section.

n/a	Confirmed	
☐	☒	The exact sample size (*n*) for each experimental group/condition, given as a discrete number and unit of measurement
☐	☒	A statement on whether measurements were taken from distinct samples or whether the same sample was measured repeatedly
☒	☐	The statistical test(s) used AND whether they are one- or two-sided<br>*Only common tests should be described solely by name; describe more complex techniques in the Methods section.*
☒	☐	A description of all covariates tested
☒	☐	A description of any assumptions or corrections, such as tests of normality and adjustment for multiple comparisons
☐	☒	A full description of the statistical parameters including central tendency (e.g. means) or other basic estimates (e.g. regression coefficient) AND variation (e.g. standard deviation) or associated estimates of uncertainty (e.g. confidence intervals)
☒	☐	For null hypothesis testing, the test statistic (e.g. *F*, *t*, *r*) with confidence intervals, effect sizes, degrees of freedom and *P* value noted<br>*Give P values as exact values whenever suitable.*
☒	☐	For Bayesian analysis, information on the choice of priors and Markov chain Monte Carlo settings
☒	☐	For hierarchical and complex designs, identification of the appropriate level for tests and full reporting of outcomes
☒	☐	Estimates of effect sizes (e.g. Cohen's *d*, Pearson's *r*), indicating how they were calculated

Our web collection on statistics for biologists contains articles on many of the points above.

Software and code

Policy information about availability of computer code

Data collection	No software was used in data collection.
Data analysis	Software used in data analysis includes: bbduk v.38.87, Sickle v1.33, SPAdes3 v.3.15.5 , Bowtie2 v 2.3.4.1, CONCOCT v0.4.0, MaxBin2 v2.2.6, MetaBAT2 v2.12.1, DAS Tool v1.1.1 , CheckM v1.0.13 , DRAM tool v1.0 , GTDB-tk r89, GToTree v1.5.38 ; and R v4.2.2 and R packages 'ggtree' v3.0.4, 'treeio' v1.16.2, 'vegan' v2.6-4, 'ggplot2' v3.4.2, and 'bit' v1.8.53.<br>Custom bash and R code used for the analyses in this manuscript has been provided for download via https://github.com/carleton-envbiotech/Methane_metagenomics.

For manuscripts utilizing custom algorithms or software that are central to the research but not yet described in published literature, software must be made available to editors and reviewers. We strongly encourage code deposition in a community repository (e.g. GitHub). See the Nature Portfolio guidelines for submitting code & software for further information.

Data

Policy information about availability of data

All manuscripts must include a data availability statement. This statement should provide the following information, where applicable:

- Accession codes, unique identifiers, or web links for publicly available datasets
- A description of any restrictions on data availability
- For clinical datasets or third party data, please ensure that the statement adheres to our policy

All data necessary to interpret, verify, and extend the research presented in this article are publicly available. The sequencing data generated in this study have been deposited to NCBI under BioProject PRJNA900590 [https://www.ncbi.nlm.nih.gov/bioproject/?term=PRJNA900590]. Within this BioProject, the raw reads files are available on the SRA database, under Biosamples SAMN31696084 – SAMN31696092. The 1,892 MAGs have been deposited to the WGS database under accessions SAMN32731718 – SAMN32731810 (STA), SAMN32731811 – SAMN32731998 (STB), SAMN32733587 – SAMN32733720 (STC), SAMN32734194 – SAMN32734413 (STD1), SAMN32734415 – SAMN32734683 (STD2), SAMN32734737 – SAMN32734946 (STE), SAMN32737191 – SAMN32737484 (STF1), SAMN32737485 – SAMN32737723 (STF2). The geochemical monitoring raw data generated in this study are provided in the Source Data file hosted on the Open Science Framework under DOI 10.17605/OSF.IO/6X5ZC – provider identification is held anonymous at the request of the landfill site engineers. The processed annotation data are provided in the Supplementary Data Files 1-7 which are hosted on the Open Science Framework under DOI 10.17605/OSF.IO/6X5ZC. Databases accessed were KEGG (https://www.genome.jp/kegg/), PFAM (https://www.ebi.ac.uk/interpro/), GTDB (https://gtdb.ecogenomic.org/), and UniRef90 (https://www.uniprot.org/help/uniref).

Research involving human participants, their data, or biological material

Policy information about studies with human participants or human data. See also policy information about sex, gender (identity/presentation), and sexual orientation and race, ethnicity and racism.

Reporting on sex and gender	N/A, there was no human participation in this study.
Reporting on race, ethnicity, or other socially relevant groupings	N/A
Population characteristics	N/A
Recruitment	N/A
Ethics oversight	N/A

Note that full information on the approval of the study protocol must also be provided in the manuscript.

Field-specific reporting

Please select the one below that is the best fit for your research. If you are not sure, read the appropriate sections before making your selection.

☒ Life sciences ☐ Behavioural & social sciences ☐ Ecological, evolutionary & environmental sciences

For a reference copy of the document with all sections, see nature.com/documents/nr-reporting-summary-flat.pdf

Life sciences study design

All studies must disclose on these points even when the disclosure is negative.

Sample size	Sample sizes were based on number of existing cells at the landfill surveyed, and the limited site access allowing one sample per cell.
Data exclusions	No data were excluded from this study.
Replication	Replication was not possible due to cost constraints for sequencing replicate samples, and site access agreements did not allow for temporal replicates.
Randomization	There were no experimental groups, and thus no randomization was required.
Blinding	There were no experimental groups and thus no blinding was required.

Reporting for specific materials, systems and methods

We require information from authors about some types of materials, experimental systems and methods used in many studies. Here, indicate whether each material, system or method listed is relevant to your study. If you are not sure if a list item applies to your research, read the appropriate section before selecting a response.

Materials & experimental systems

n/a	Involved in the study
☒	☐ Antibodies
☒	☐ Eukaryotic cell lines
☒	☐ Palaeontology and archaeology
☒	☐ Animals and other organisms
☒	☐ Clinical data
☒	☐ Dual use research of concern
☒	☐ Plants

Methods

n/a	Involved in the study
☒	☐ ChIP-seq
☒	☐ Flow cytometry
☒	☐ MRI-based neuroimaging
